# Aqueous Extract of Pepino (*Solanum muriactum* Ait) Leaves Ameliorate Lipid Accumulation and Oxidative Stress in Alcoholic Fatty Liver Disease

**DOI:** 10.3390/nu10070931

**Published:** 2018-07-20

**Authors:** Jen-Ying Hsu, Hui-Hsuan Lin, Cheng-Chin Hsu, Bing-Chen Chen, Jing-Hsien Chen

**Affiliations:** 1 Department of Nutrition, Chung Shan Medical University, No. 110, Sec. 1, Jianguo N. Road, Taichung City 40201, Taiwan; jyhsu0530@gmail.com (J.-Y.H.); king@csmu.edu.tw (C.-C.H.); eddie790329@gmail.com (B.-C.C.); 2 Department of Medical Laboratory and Biotechnology, Chung Shan Medical University, No. 110, Sec. 1, Jianguo N. Road, Taichung City 40201, Taiwan; linhh@csmu.edu.tw; 3 Department of Clinical Laboratory, Chung Shan Medical University Hospital, No. 110, Sec. 1, Jianguo N. Road, Taichung City 40201, Taiwan

**Keywords:** aqueous extract of pepino leaf, alcoholic fatty liver disease, anti-inflammation, oxidative stress, functional foods

## Abstract

Chronic alcohol intake leads to alcoholic fatty liver. The pathogenesis of alcoholic fatty liver is related to abnormal lipid accumulation, oxidative stress, endotoxins, and cytokines. *Solanum muricatum* Ait. (Pepino) is a plant food commonly cultivated in the Penghu island, Taiwan. Previous studies indicated that the aqueous extract of pepino was able to attenuate diabetic progression via its antioxidative and anti-inflammatory effects. However, the mechanisms of the antioxidative and anti-inflammatory effects of pepino leaf in preventing alcoholic fatty liver remain unknown. In this study, Lieber–DeCarli ethanol-containing liquid diet was used to induce alcoholic hepatic injury in C57BL/6 mice. The hepatoprotective effects and the related mechanisms of aqueous extract of pepino leaf (AEPL) were examined. Our results showed that 2% AEPL treatments protected the liver from ethanol-induced injury through reducing serum levels of aspartate aminotransferase (AST), alanine aminotransferase (ALT), total cholesterol (TC) and triglyceride (TG) (all *p* < 0.05). AEPL had the effects in improving the ethanol-induced lipid accumulation in mice under histological examination. Molecular data indicated that the anti-lipid accumulation effect of AEPL might be mediated via inducing hepatic levels of phospho-adenosine monophosphate-activated kinase (p-AMPK) and peroxisome proliferator-activated receptor (PPAR)-α, and reducing the expressions of hepatic lipogenic enzymes, including sterol regulatory element-binding protein (SREBP)-1c, acetyl-CoA carboxylase (ACC), and fatty acid synthase (FAS) (all *p* < 0.05). AEPL also decreased hepatic levels of thiobarbituric acid relative substances (TBARS), tumor necrosis factor (TNF)-α, and interleukin (IL)-6, as well as the expression of nuclear factor kappa B (NF-κB) (all *p* < 0.05). Moreover, AEPL significantly elevated the activities of superoxide dismutase (SOD), catalase, and glutathione peroxidase (GPx), and glutathione (GSH) content compared to the ethanol-fed group (all *p* < 0.05). Our present study suggests that AEPL could protect the liver against ethanol-induced oxidative injury and lipid accumulation.

## 1. Introduction

Alcoholic fatty liver, also called liver steatosis, is the earliest response to chronic alcohol abuse and is one of the stages of the alcoholic liver disease. Over 90% of the heavy drinkers develop alcoholic fatty liver [1] which is a major risk of liver fibrosis, hepatic cirrhosis and carcinoma [2]. Alcoholic fatty liver is considered to be a vicious circle due to abnormal lipogenesis and lipid β-oxidation. According to the previous study, long-term alcohol exposure would increase lipogenesis related with AMP-activated protein kinase (AMPK) activity, sterol regulatory element binding proteins (SREBPs), acetyl-CoA carboxylase (ACC), and fatty acid synthase (FAS) [3]. Peroxisome proliferator-activated receptors (PPAR)-α is a key element of fatty acid catabolism in the liver as PPAR-α regulates many target genes expression including carnitine palmitoyltransferase 1 (CPT-1) [4]. CPT-1 is a transporter protein located in mitochondria and its function is to control fatty acid transporting into mitochondria for β-oxidation [4]. However, long-term alcohol exposure inhibited lipid β oxidation as both PPAR-α and CPT-1 activity are disturbed [1]. These findings suggest that PPAR activation is a promising therapeutic strategy in the alcoholic fatty liver.

Oxidative stress comes from intracellular reactive oxygen species (ROS) [5]. Alcohol in liver is metabolized by alcohol dehydrogenase (ADH) and cytochrome P450 2E1 (CYP 2E1). Both metabolism process and metabolite (acetaldehyde) are the major resources of ROS [2,6]. For alcohol is one of the inducers of CYP 2E1, chronic alcohol consumption would enhance CYP 2E1 expression and result in more ROS production and oxidative stress [7]. Overwhelming oxidative stress causes cellular damage such as lipid peroxidation occurred [8]. Lipid peroxidation forms malondialdehyde (MDA) and 4-hydroxynonenal (HNE) that both possess hepatotoxicity [9]. The antioxidant factors for balancing intracellular oxidative stress can be divided into two types: enzyme type including superoxide dismutase (SOD), catalase, and glutathione peroxidase (GPx), and non-enzyme type, such as glutathione (GSH) [5]. Based on the past study, chronic alcohol consumption declined the content of SOD, catalase, GPx, and GSH [10]. Elevating antioxidant capacity could effectively protect hepatocytes from the alcohol-induced liver injury [11]. Inflammatory cytokines also induce oxidant formation and cause liver injury. Chronic alcohol consumption increases gut permeability thus leads LPS to translocate from intestinal to liver. Enteric LPS was recognized by toll-like receptor 4 (TLR 4) on the kupffer cell surface and stimulated the release of inflammatory cytokines [12].

Phytochemicals, contained in many herbs, have been revealed with various protective effects for alcohol-induced liver injury [10], such as lemon juice, mulberry, *Ganoderma lucidum*, and *Scutellaria baicalensis* Georgi, showed multiple protective effects for preventing the progression of alcoholic fatty liver [11,13,14,15]. Several studies demonstrated that phytochemicals which elevate antioxidant capacity would protect hepatocytes [10,11,14]. The use of phytochemicals in alcoholic fatty liver treatment could avoid drug adverse effects as well as decrease medical costs. *Solanum muriactum* Ait, also called pepino, is mainly cultivated in Peng Hu, Taiwan. In the past studies, pepino fruit has been demonstrated to have anti-tumor, antioxidative, anti-inflammatory, and antiglycative effects [16,17]. Its aqueous extract was able to improve oxidative and glycative stress to protect sciatic nerve in diabetic mice [18]. After harvesting pepino fruit, pepino leaves are mostly discarded or fed to goats in local. As far as we know, there is no relevant research about the biological effect of pepino leaves, including the protective effects against alcoholic fatty liver disease. So, this has great potential for research. In this study, we used the aqueous extract of pepino leaves (AEPL) to investigate how pepino leaves affect the progression of alcoholic fatty liver, including lipid accumulation, anti-inflammation, and antioxidant capacity in the chronic alcohol consumption animal model. Once the intervention of AEPL could be evidenced to improve alcoholic fatty liver, these findings would bring benefits to alcoholic fatty liver treatment as well as raise the economic value of pepino leaves and reduce medical costs.

## 2. Materials and Methods 

### 2.1. Preparation of AEPL and Alcohol-Containing Liquid Diet

Dried pepino (*Solanum muricatum* Ait.) leaves were obtained from the farm in Peng Hu Island, Taiwan. The stem of the pepino leaves should be removed before extraction. A 100 g pepino leaves were boiled and simmered in 4 liters of deionization water for 2 h. After cooling to room temperature, the aqueous extract was filtered through Advantec No. 1 filter paper (Toyo Roshi Kaisha, Ltd., Tokyo, Japan). The remnant leaves were mixed with 2 liters of deionization water and repeated the extraction process. The filtrate was further freeze-dried to a fine powder. The extraction yield of pepino leave was about 18%. We quantified total phenolic acids, flavonoids, and anthocyanins in AEPL as shown in Table S1. To confirm the reproducibility of extraction, we quantify the above ingredients before using in the experiment. The alcohol-induced liver injury was induced by Lieber-DeCarli alcohol-containing liquid diet made from micro-stabilized rodent liquid diet (LD 101A; Test-Diet, Richmond, IN, USA). The calories of carbohydrate, protein, and fat in liquid diet were 12%, 17%, and 35% of total calories, respectively. Alcohol contained in the liquid diet provided 36% of total calories (50.9 g/L). Maltodextrin (90 g/L) was replaced by the calorie of alcohol in a normal liquid diet. For experimental diet, a 140 g liquid diet was blended 1% or 2% dose of AEPL and mixed with deionization water for 1 kcal/g. The composition and calories contained in one-kilogram liquid diet was shown in Table 1.

### 2.2. Animals and Experimental Design

Male C57BL/6J mice, 5 weeks old, were obtained from National Laboratory Animal Center (National Science Council, Taipei City, Taiwan). The use of mice was reviewed and approved by Chung Shan Medical University Animal Care Committee (IACUC approval number: 1642). Mice were housed in a constant condition of temperature (22 ± 2 °C) and humidity (55 ± 2%) room on a 12-h light/dark cycle. After adaptation of the environment, the mice were randomly divided into four groups (ten mice per group) and treated as follows: control group (maltodextrin–containing liquid diet group), EtOH group (alcohol-containing liquid diet group), EtOH + 1% AEPL, and EtOH + 2% AEPL. EtOH group and AEPL groups were fed Lieber–DeCarli alcohol-containing liquid diet since 1st week. The dose of alcohol for 3 groups was gradually increased 10% of total energy on day 1 and 2, 20% on day 3 and 4, 30% on day 5 and 6, and on 36% on day 7 and thereafter. All groups were fed for 5 weeks. During the experiment period, all mice were given access to food and water ad libitum. Body weight was recorded every week. At the end of the experiment, mice were euthanized by carbon-dioxide asphyxiation followed by exsanguination. The liver samples and blood were collected and used for further experiments.

### 2.3. Serum Biochemical Parameters

Blood samples were centrifuged at 3000 rpm to separate serum and stored at 4 °C for determining serum biochemistry parameters. The serum levels of aspartate aminotransferase (AST), alanine aminotransferase (ALT), total cholesterol (TC), triglyceride (TG), HDL (high-density lipoproteins), LDL (low-density lipoproteins), and VLDL (very-low-density lipoproteins) were determined by the medical laboratory in Chung Shan Medical University Hospital. 

### 2.4. Measurement of Thiobarbituric Acid Relative Substances (TBARS) and Antioxidant Status in Liver 

The lipoperoxide level in liver tissues was measured by thiobarbituric acid reactive substances (TBARS, nmol/mg protein). TBARS assay was performed and modified according to the procedures of Ohkawa et al., (1976) [19]. Quantification of TBARS was determined by using fluorescence spectrophotometer (excitation at 532 nm and emission at 600 nm) and comparison with a standard curve of malondialdehyde (MDA), the lipid peroxidation product, which is generated by acid-catalyzed hydrolysis of 1,1,3,3-tetramethoxypropane. 

The procedure used for determining GSH with 5-thio-2-nitrobenzoic acid (TNB) was performed as previously described [20]. TNB-derived absorption was measured at 405 nm (VERSA max B02153). GPx activity was determined spectrophotometrically according to the method of Knight SA, Sunde RA [21]. The enzyme activity was calculated by the change of the absorbance value at 340 nm for 3 min. The SOD assay was conducted using a modified and described previously [22]. The samples were measured at the absorption wavelength of 450 nm in a spectrophotometer. Catalase activity was measured based on the ability of the enzyme to break down H_2_O_2_ according to a modification of the method proposed by Aebi (1974) [23]. The samples were measured at an excitation wavelength of 240 nm for 1 min in a spectrophotometer.

### 2.5. Trolox Equivalent Antioxidant Capacity (TEAC) Assay

The TEAC assay was carried out according to the procedure in the literature [24]. Briefly, the ABTS^∙+ ^ stock solution was prepared from 44 U/mL peroxidase, 750 µM ABTS solution, and 500 µM hydrogen peroxide and then placed in the dark for 6h at room temperature. The diluted sample was mixed with 0.5 mL of ABTS, and the absorbance of the mixture was measured at 734 nm after 6 min, and the percent of inhibition of absorbance at 734 nm was calculated. The reference standard was Trolox, and the results were expressed as mM Trolox/mg protein.

### 2.6. Measurement of Liver TG Levels

To quantify the liver TG level, liver tissues were homogenized in RIPA (radioimmunoprecipitation assay) lysis buffer (50 mM Tris-HCl + 1% NP-40 + 0.25% Na-deoxycholate + 150 mM NaCl + 1 mM EDTA, pH 7.4) and centrifuged. Liver TG was measured by using Triglycerides colorimetric assay kit (Cayman Chemical Company, Ann Arbor, MI, USA) according to the manufacturer’s instructions.

### 2.7. Measurement of Liver Tumor NecrosisFfactor (TNF)-α and Interleukin (IL)-6 Levels

The levels of TNF-α and IL-6 in liver tissues were assayed by ELISA MAX™ Deluxe set (BioLegend, San Diego, CA, USA) on the basis of manufacturer’s protocols. The absorbance was measured at 450 nm in a microplate reader (VersaMax Tunable Microplate reader Molecular Devices, Molecular Devices Co., Sunnyvale, CA, USA).

### 2.8. Histological Evaluation

At the end of the experiment, all groups were sacrificed and collected the right lobe of liver for histological evaluation. Liver histological assessments were performed in 2 methods, hematoxylin and eosin (H&E) staining and Nile red staining. For the H&E staining, pieces of liver were fixed in 10% buffered neutral formalin, followed by dehydration with different concentrations of ethanol and xylene, and then replacing xylene with hot paraffin. Finally, the tissue was embedded in paraffin solution. The paraffin sections were prepared and cut into 5 µm thick sections using a sliding microtome (Pteratome CMR440, Sakura, Tokyo, Japan). The sections were passed through xylene, alcohol, water, and then stained with H&E dye. Nile red staining was modified by Fowler and Greenspan (1985) [25]. Frozen sections 10 µm thickness were stained in Nile red 2 µg/mL in 75% glycerol and mounted with coverslips for 5 min. The sections were examined by confocal microscopy.

### 2.9. Protein Preparation and Western Blot Analysis 

Proteins from liver tissues were homogenized on ice in 1 mL RIPA lysis buffer containing a protease inhibitor cocktail (Pierce; Thermo Fisher Scientific, Rockford, IL, USA). Homogenized samples were subjected to centrifugation at 4 °C, 12,000 rpm for 10 min. The total protein concentration was determined by the BCA (bicinchoninic acid) protein assay kit (Pierce; Thermo Fisher Scientific, Rockford, IL, USA). Total protein (10–50 µg per lane) was electrophoresed and separated by 8% or 10% SDS-poly-acrylamide gels and transferred to nitrocellulose membranes (Whatman, GE Healthcare, Freiburg, Germany). The membranes were blocked with 5% non-fat milk and then incubated with the indicated primary antibody overnight at 4 °C. The blots were incubated with the antibodies against p-AMPK, AMPK, PPAR-α, CPT-1, SREBP-1, FAS, ACC, p-ACC (phospho-acetyl-CoA carboxylase), CYP2E1, TLR 4, NF-κB and β-actin, purchased from Santa Cruz Biotechnology, Inc. (Santa Cruz, CA, USA). The blot was quantified by enhanced chemiluminescence detection (Amersham Pharmacia Biotech, Little Chalfont, Bucks, UK). Quantification of the bands was carried out by measuring the intensity of the immunoblot band using ImageQuant™ LAS 4000 mini (GE Healthcare Bio-Sciences AB, Uppsala, Sweden). β-actin is an internal control for ensuring the loading is equal.

### 2.10. Statistical Analysis

The effect of each treatment was analyzed from 10 mice (*n* = 10) in each group. Data were expressed as mean ± standard deviation (SD). Statistical analysis was done using one-way ANOVA (analysis of variance), and post hoc comparisons were carried out using Duncan’s multiple-range test. Differences with *p* < 0.05 were considered to be significant. 

## 3. Results

### 3.1. AEPL Helped Maintain Mice Body Weight

Mice body weight was weighted every week during experiment period. The changes of mice body weight were shown in Table 2. During the treatment period, the body weight in mice in EtOH group (21.0 ± 0.8 g) was significantly lower than that of the control group (23.0 ± 0.6 g) since the 3rd week. The body weight of mice in both groups of EtOH + 1% AEPL (22.5 ± 0.6 g) and EtOH + 2% AEPL (22.0 ± 0.9 g) in 3rd and 4th week was not significantly different from that of the control group but was significantly higher than EtOH group. At the 5th week, the mice in EtOH + 1% AEPL group had significantly lower body weight than the control group, but that was still significantly higher than EtOH group. These results indicated that feeding alcohol-containing liquid diet to mice might cause weight loss. However, the addition of AEPL in diet could contribute to maintaining mice body weight, especially with a high dose of AEPL. At the end of the experiments, all mice were euthanized and liver samples were collected and weighted. The changes in liver weights in each group were shown in Table 2. There was no significance in 1% or 2% AEPL group compared with the control group or EtOH group.

### 3.2. AEPL Diminished Serum AST, ALT, TC, TG, LDL, and VLDL, and Hepatic TG Level

AST and ALT are important biomarkers of liver function. The elevation of AST and ALT is a critical clinical sign of injured hepatocytes. After we fed alcohol-contained liquid diet for 5 weeks, AST and ALT were increased in EtOH group as compared with control group. Nevertheless, AST and ALT decreased by 1% or 2% AEPL-treatment group. To investigate the effect of AEPL on lipid content, serum lipid content and liver TG content were analyzed and showed in Table 3. After 5-weeks of alcohol treatment, serum TC, TG, HDL, LDL, and VLDL in EtOH group were significantly higher than those of the control group. Comparing to EtOH group, serum TG, LDL, and VLDL in 1% AEPL group reduced 21%, 27%, and 19%, respectively. And in 2% AEPL group, serum cholesterol, TG, LDL, and VLDL were lower 22%, 22%, 20%, and 31% than in EtOH group. The decline of serum cholesterol content in 1% or 2% AEPL group showed a dose-dependent manner. Serum HDL content in 1% or 2% AEPL group was higher than control group and similar with EtOH group. We also determined the TG content in liver. In EtOH group, the TG content was significantly higher than other groups. While in 1% and 2% AEPL groups, the liver TG content was lower than EtOH group in a dose-dependent manner. These results demonstrated that the 1% and 2% AEPL treatment would preserve liver function, decrease hyperlipidemia, and reduced TG accumulation in the liver.

### 3.3. AEPL Attenuated Alcohol-Induced Liver Lipid Accumulation Via Histological Assessment

**Figure 1:**
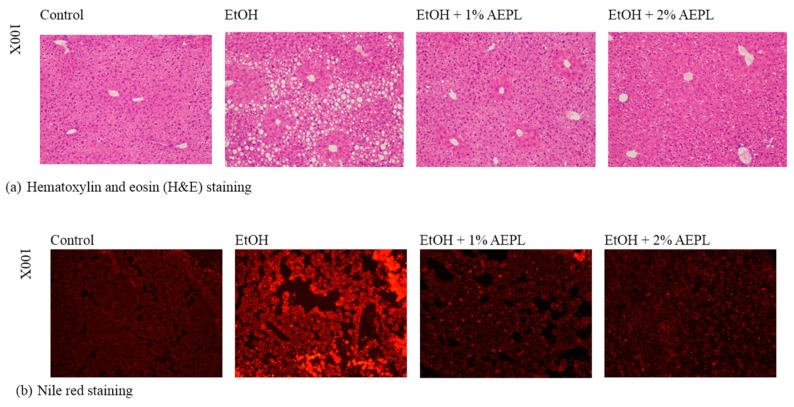
AEPL inhibited chronic alcohol-induced lipid accumulation in mice liver. Morphological examination of hematoxylin and eosin (H & E) stained (**a**) and Nile red stained (**b**) livers from control, EtOH-treated, EtOH + 1% AEPL and EtOH + 2% AEPL mice at week 5.

The liver histological assessment was applied for the investigation of hepatic lipid accumulation in mice liver. H&E stain of the liver sections showed massive steatosis in EtOH group, which was obviously reduced in 1% or 2% AEPL group to a level similar to that of the control group (Figure 1a). We also observed the infiltration of inflammatory cell in some of the slides of EtOH group. However, such a situation was not observed in AEPL group. The lipid-specific staining was performed by Nile red stain. Nile red was a hydrophobic lipid probe. As shown in Nile red-stained liver section, the distribution of neutral lipids was improved in 1% or 2% AEPL group (Figure 1b). Basing on the results presented above we demonstrated that chronic alcohol feeding combined with 1% or 2% AEPL could suppress lipid accumulation in the liver. 

### 3.4. AEPL Improved the Lipid Metabolism by Lipid Synthesis and Oxidation 

**Figure 2:**
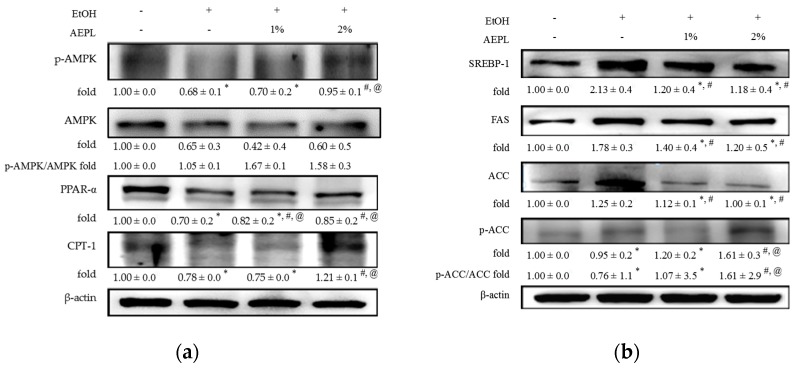
AEPL increased protein levels of p-AMPK, AMPK, PPAR-α, and CPT-1, and decreased protein levels of SREBP-1, FAS, p-ACC, and ACC. (**a**) Protein levels of p-AMPK, AMPK, PPAR-α, and CPT-1. (**b**) Protein levels of SREBP-1, FAS, ACC, and p-ACC. Data were presented as mean ± SD from at least 3 independent experiments and expressed as the percentage of the control. +/− indicates whether the diet contains with /without ethanol or AEPL. * *p* < 0.05, compared with control group; ^#^ *p* < 0.05, compared with ethanol group; ^@^ *p* < 0.05, compared with EtOH + 1% AEPL group. p-AMPK: phospho-adenosine monophosphate-activated kinase; AMPK: AMP-activated protein kinase; PPAR-α: Peroxisome proliferator-activated receptors; CPT-1: carnitine palmitoyltransferase 1; SREBP-1: sterol regulatory element-binding protein; FAS: fatty acid synthase; ACC: acetyl-CoA carboxylase; p-ACC: phospho-acetyl-CoA carboxylase.

In order to further understand the effect of AEPL on lipid metabolism in the liver, we analyzed the levels of p-AMPK, PPAR-α, CPT-1, SREBP-1, FAS, ACC, and p-ACC. The levels of p-AMPK, PPAR-α, and CPT-1 were reduced in EtOH group as compared with control group (Figure 2a). The protein levels of p-AMPK in 1% or 2% AEPL raised, dose-dependently, 7%, and 84% respectively as compared with EtOH group. The levels of PPAR-α and CPT-1 in 2% AEPL receptively raised 50% and 21% as compared with EtOH group (Figure 2a). As presented in Figure 2b, EtOH group had significant increases in levels of SREBP-1 by 113% and FAS by 78% as compared with control group, which would lead to an activation of lipid synthesis pathway in the liver. Whereas the levels of SREBP-1 and FAS showed a great reduction in the presence of 1% or 2% AEPL that were similar to those of control group. Kim et al., (1989) have indicated that changes in the protein level of ACC in many tissues have been related to alterations in the activity of ACC. It further showed that the inhibition of ACC activation is mediated by increasing ACC phosphorylation [26]. In agreement with the findings, the data in Figure 2b demonstrated that the protein level of ACC, which are recognized as the ACC activation, was increased in liver tissues of EtOH group, but those of p-ACC were charged slightly. It was further found that AEPL treatments dose-dependently reduced the protein level of ACC in liver tissues, whereas AEPL treatments at 2% significantly induced about 1.4-fold of p-ACC compared with EtOH group. There was also the same trend for the ratio of p-ACC/ACC. These results suggested that AEPL could reverse lipid synthesis and oxidation in the liver through regulating enzymes and transcription factors.

### 3.5. AEPL Could Suppress the Expression of Liver Cytochrome P 2E1 

**Figure 3:**
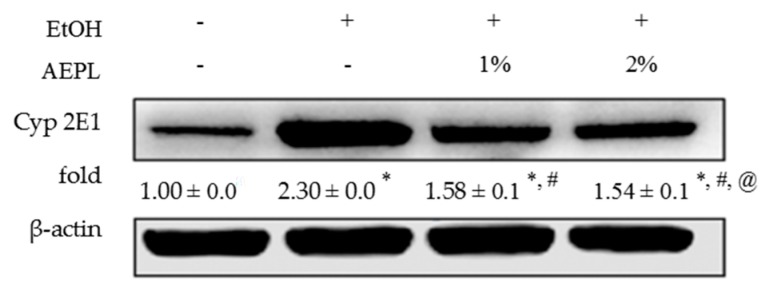
AEPL reduced protein level of CYP 2E1. Data were presented as mean ± SD from at least 3 independent experiments and expressed as the percentage of the control. +/− indicates whether the diet contains with/without ethanol or AEPL. * *p* < 0.05, compared with control group; ^#^ *p* < 0.05, compared with ethanol group; ^@^ *p* < 0.05, compared with EtOH + 1% AEPL group.

CYP 2E1 metabolizes alcohol and produces acetaldehyde and ROS. At the same time, alcohol is an inducer of CYP 2E1. We hypothesized that the intervention of AEPL would reduce CYP 2E1. As expected, CYP 2E1 expression was significantly elevated by 1.3 fold in EtOH group than in control group (Figure 3). While the level of CYP 2E1 in 1% or 2% AEPL treatment group was significantly lower than EtOH group by 28% and 33%, respectively. However, while comparing to control group, CYP 2E1 expression in 1% or 2% AEPL treatment group were obviously higher by 58% and 54%, respectively. Basing on the results, AEPL would suppress the CYP 2E1 expression which was induced by alcohol.

### 3.6. AEPL Reduced Alcohol-Induced Inflammatory Cytokines Levels 

**Figure 4:**
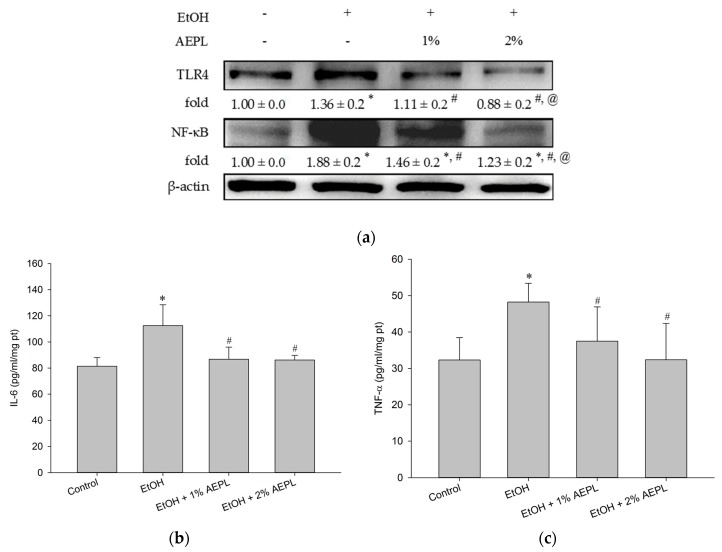
Effect of alcohol and 1% or 2% AEPL on the protein levels of TLR 4, NF-κB, IL-6 and TNF-α. (**a**) The protein level of TLR 4 and NF-κB. (**b**) The level of IL-6 in liver. (**c**) The level of TNF-α in liver. +/− indicates whether the diet contains with/without ethanol or AEPL. Data were presented as mean ± SD from at least 3 independent experiments, and expressed as the percentage of the control. * *p* < 0.05, compared with control group; ^#^ *p* < 0.05, compared with ethanol group; ^@^ *p* < 0.05, compared with EtOH + 1% AEPL group.

According to the past studies, long-term alcohol consumption would increase enteric permeability that enables enteric endotoxin to translocate from intestine to liver. Endotoxin would be recognized by TLR 4 and produce the intracellular signal to activate NF-κB to produce and release more inflammatory cytokines. In our study, TLR 4 protein level in EtOH group was significantly higher than the control group, whereas 1% or 2% AEPL down-regulated the expression of TLR 4 in a dose-dependent manner (Figure 4a). The level of NF-κB in 1% or 2% AEPL group was also significantly lower than EtOH group (Figure 4a). The changes of IL-6 and TNF-α in each group were presented in Figure 4b,c. The expression of TNF-α and IL-6 in EtOH group was significantly higher than the control group. In 1% or 2% AEPL group, the expression of TNF-α and IL-6 was both lower than EtOH group and restored back to normal concentration.

### 3.7. AEPL Increased Antioxidant Substances and Decreased Lipid Peroxidation 

Alcohol exposure induces hepatocytes oxidative stress formation and leads to lipid peroxidation. In addition, excess alcohol consumption also declines antioxidant capacity. We suspected that AEPL would improve antioxidant capacity, and thus we analyzed the contents of SOD, catalase, GPx, and GSH. In EtOH group, the contents of SOD, catalase, and GPx were lower than the control group. Though GSH level in EtOH group was higher than the control group, there was no significance compared with control group. This may be concerned with the variance within EtOH group. TEAC in EtOH group was also reduced as compared to control group (Table 4). SOD, catalase, GPx, and GSH were increased in both 1% and 2% AEPL groups (Table 4). The content of catalase and GPx, in particular, showed dose-dependent responses. In 2% AEPL group, not only SOD, catalase, and GPx were recovered to normal levels, but also has a significant increase in GSH level compared with the control group and TEAC was reversed to control level. 

If overwhelm oxidative stress cannot be neutralized by antioxidant substances, organelles, including lipid, proteins, and DNA, would suffer from oxidative damage. Lipid peroxidation is a kind of oxidative damage process and produces reactive and hepatotoxicity metabolites, MDA, and 4-HNE [9]. In other words, the variation amount of MDA may reflect the degree of oxidative stress. The present study explored whether the intervention of AEPL with different doses would decrease the lipid peroxidation which was elevated after continuous alcohol consumption. The result was showed in Table 4 and presented as the content of MDA. The higher the content of MDA means the higher extent of lipid peroxidation. The content of MDA in EtOH group was significantly higher by 1.6 folds than control group. Nevertheless, MDA content in the 1% or 2% AEPL group were significantly lower by 76% than alcohol group, and similar to the control group. The results indicated that the lipid peroxidation induced by alcohol consumption was repressed through the intervention of AEPL.

## 4. Discussion

Though the pathologic progression of alcoholic fatty liver includes multi-factors, the prevention or protection of alcohol-induced liver injury should be multi-oriented. In the present study, we provided Lieber-DeCali liquid nutrient diet to mice to establish chronic alcohol consumption model that has been widely used as model systems in mimicking human patterns of chronic alcohol consumption [27]. AEPL (1% or 2%) was blended with alcohol-contained liquid diet in experimental groups to investigate whether the intervention of low or high dose of AEPL would protect hepatocytes and prevent fatty liver progression.

According to our results, we observed that the weight loss in EtOH group but recovering in AEPL groups. The weight loss in EtOH group might be related with energy wastage. It has been reported that deterioration of energy wastage associated with chronic alcohol intake and CYP 2E1-mediated metabolism [28]. However, the mechanism of energy wastage is not clear [29]. In our results, the intervention of AEPL decreased CYP 2E1 activity which might improve energy wastage status. In addition, during the experiment, we changed the liquid diet every day and gave the same weight to EtOH group and AEPL groups. However, the liquid diet of 1% and 2% of AEPL groups were eaten completely, but the EtOH group still be left. The weight gain of mice in the AEPL groups may due to the increase of food intake or other factors that promote appetite in mice. Further analysis will be applied in the future.

In ethanol group, serum TC, TG, LDL, and VLDL were significantly higher than in control group. Liver TG was also marked accumulation in ethanol group. However, in the intervention of 1% or 2% AEPL treatment, both serum TC, TG, LDL, and VLDL, and hepatic TG were significantly reduced. The histological changes could more clearly be observed that lipid droplets and its accumulation were attenuated in AEPL groups. We thus further analyzed the possible mechanism.

The changes of p-AMPK and SREBPs activity lead to abnormal lipid synthesis in liver. In our results, the expression of p-AMPK was suppressed but increasing SREBP-1 level in EtOH group. ACC and FAS level, the target proteins of SREBP-1, were significantly higher than the control group. However, both low and high dose of AEPL could alter the vicious circle of lipid synthesis through increasing p-AMPK level and suppressed SREBP-1, concurrently, down-regulating the expression of ACC and FAS. In addition, p-ACC level, the inactivated form of ACC and which is regulated by AMPK [3], was increased in both AEPL group. Based on our results, AEPL could cease the progression of the alcoholic fatty liver through abnormal lipid synthesis. Our study also showed that the serum cholesterol level in both doses of AEPL groups recovered to normal in a dose-dependent manner. According to the past studies, hypercholesterolemia or liver cholesterol accumulation were thought to be related to SREBP-2 and regulated by HMG-CoA reductase, which is the rate-limiting enzyme in cholesterol synthesis pathway [30,31]. However, further investigation is needed to clarify whether the reduction of serum cholesterol level by AEPL is concerned with SREBP–2. 

Chronic alcohol exposure disturbs not only lipid synthesis but also lipid oxidation. The activity of PPAR-α is affected by chronic alcohol consumption for down-regulating AMPK phosphorylation [32,33]. Consistent with previous studies, PPAR-α expression, as well as CPT-1 level, significantly declined in EtOH group. However, both low and high doses of AEPL increased PPAR-α expression and CPT-1 level. According to Monika Fischer research, giving PPAR-α agonist to alcohol-fed mice induced the mRNA level of PPAR-α targeted gene and resulted in a high rate of fatty acid β-oxidation, normalization of serum TG levels, and a reduction of TG accumulation in the liver [34]. As the previous study, we found both serum TG level and liver TG accumulation significantly declined in AEPL treated mice. The results may be indicated that the components of AEPL possess PPAR-α agonist activity. Further AEPL component analysis is required. Our findings demonstrate that AEPL could improve both lipid synthesis as well as oxidation to decrease lipid accumulation in the liver and serum lipid profile. Our results found the serum HDL level in ethanol-fed groups were higher than the control group. The previous study revealed that moderate alcohol-intake could increase serum HDL level. The mechanism may be concerned with reducing CEPT activity or increasing transport rate of apolipoproteins A-I and A-II or the activity of ALDH2 [35,36,37]. Therefore, the actual mechanism of how AEPL effect on HDL is still needed to be clarified further.

Chronic alcohol consumption causes hepatocyte oxidative stress formation and depletes antioxidant substances, thus leads to hepatocytes injury. ADH and CYP 2E1-mediated alcohol metabolism and inflammation cytokines are the major sources of ROS leading to oxidative injury in hepatocytes [8,12]. According to our results, both doses of AEPL decreased liver CYP 2E1 expression, TLR4 level, and IL-6 and TNF-α level. Besides, AEPL in low or high dose recovers MDA to the normal level. These results indicated that AEPL could decline the alcohol-induced intracellular oxidative stress. While in antioxidant capability, our results showed that AEPL, especially in high dose, restored SOD, catalase, GPx, and TEAC as well as increased GSH content. Overall, AEPL not only reduced oxidative stress formation but also increased antioxidant capacity to protect hepatocyte from alcohol-induced injury.

Inflammation has a pivotal role in alcohol-induced liver injury as the source of oxidative formation [38]. According to the past research, chronic alcohol consumption led to increased gut permeability and made enteric endotoxin translocate to the liver that was recognized by TLR 4 [39,40]. TLR 4-mediated responses would induce NF-κB activation, the key transcription factor that triggers pro-inflammatory cytokine production, such as TNF-α, IL-6, IL-1β, and other chemokines [12,41]. The previous study showed that the facilitation of NF-κB stability contributed to the reduction of inflammatory cytokine expression [42]. In our study, we found that AEPL not only decreased NF-κB level but also the levels of TNF-α and IL-6. Some studies mentioned that TNF-α and IL-6 in the liver would maintain in certain concentration under the normal physiological state for hepatocytes regeneration [43,44,45]. Our study showed that AEPL restored liver levels of TNF-α and IL-6, which probably could repair hepatocytes injury and remain the ability of regeneration. We also found that the expression of TLR 4 reduced in both doses of AEPL. However, whether this result represented that AEPL could improve intestinal penetration and reduce the migration of enteric-LPS needs further investigation.

It is well established that dietary polyphenolic compounds play important roles in the prevention of fatty liver [46]. Polyphenolic compounds affect the development of fatty liver not only through modulation of hepatic lipids but also by influencing the immune and physiological processes associated with the development of this disease. In contrast with both the aqueous extracts from the leaf (AEPL) and fruits (known as pepino aqueous extract (PAE)) of pepino, the total flavonoids content of AEPL and PAE was estimated about 4550 mg [17] and 875 mg/100 g dry weight (Table S2) respectively according to the Jia method. Total anthocyanins in leaves are 2-folds higher than that of fruits according to the Fuleki and Francis method. As observed in the present investigation, AEPL and PAE had similar levels of total phenolic acids, but AEPL had a higher content of total flavonoids and anthocyanins than the PAE. The advantage of polyphenols composition should contribute indispensably for AEPL, which has been demonstrated on two aspects: (i) antioxidation, and (ii) molecular mechanisms related to anti-inflammation [46,47]. As shown in Table S1, AEPL is mainly composed of polyphenols, which are thought to contribute to their biological properties. The comparison of AEPL with various plant-derived polyphenols was shown in Table S3. Previous studies have indicated that polyphenolic compounds such as tea catechins may exert their effects through modulation of ROS, adhesion molecules, cytokines, and interaction of immune cells with endothelial cells [48]. In addition, supplementation of tea catechins has been demonstrated to decrease liver enzymes in patients with nonalcoholic fatty liver disease (NAFLD) [49]. Anthocyanins have drawn increasing attention because of their preventive effect against various diseases. Zhu et al., (2012) demonstrated that anthocyanin cyanidin-3-O-b-glucoside (C3G) treatment lowers hepatic lipid peroxidation, inhibits the release of proinflammatory cytokines, and protects against the development of hepatic steatosis [47]. The evidence cooperatively demonstrated that the hepatoprotective effect of AEPL may be performed mainly by the polyphenol component.

## 5. Conclusions

In the present study, we investigated pepino leaves to reduce alcohol-induced fatty liver. As shown in the results, the intervention of AEPL on chronic alcohol consumption C57BL/6 mice, including (i) inhibiting abnormal lipid synthesis; (ii) increasing lipid β-oxidation; (iii) attenuating inflammation cytokines TNF-α and IL-6 levels; (iv) reduced CYP 2E1 expression to inhibit oxidative stress generation and elevated antioxidant capacity for resisting oxidative stress. These results revealed evidence that pepino leaves possessed hepatoprotective effects and capability in the inhibition of alcoholic fatty liver progression. Therefore, we considered that pepino leaves possess great potential to become a functional food for alcoholic fatty liver disease in the future.

## Figures and Tables

**Table 1:** The compositions of control vs. alcohol liquid diet (one-kilogram liquid diet).

Diets Composition	Control Liquid Diet (%)	Alcohol-Containing Liquid Diet (%)
Maltodextrin	9.00	0.00
Ethanol	0.00	5.10
Carbohydrate	5.35	5.35
Protein	4.06	4.06
Fat	3.90	3.90
Fiber	0.69	0.69
Water	77.00	80.90

**Table 2:** Effects of different doses of aqueous extract of pepino leaves (AEPL) on changes in body weight during the 5-week experimental period and liver weight in alcohol-induced liver injury in mice.

Body Weight (g)				
Group Week	Control	EtOH	EtOH + 1% AEPL	EtOH + 2% AEPL
1st week	22.5 ± 0.6 ^a^	22.5 ± 0.8 ^a^	22.0 ± 0.7 ^a^	22.0 ± 0.7 ^a^
2nd week	22.5 ± 0.5 ^a^	22.5 ± 0.7 ^a^	22.5 ± 0.8 ^a^	22.5 ± 0.7 ^a^
3rd week	23.0 ± 0.6 ^a^	21.0 ± 0.8 ^b^	22.5 ± 0.6 ^a^	22.0 ± 0.9 ^a^
4th week	23.5 ± 0.9 ^a^	20.5 ± 0.5 ^b^	22.0 ± 0.8 ^a^	22.5 ± 0.9 ^a^
5th week	25.0 ± 0.7 ^a^	20.0 ± 0.7 ^c^	21.5 ± 0.9 ^b^	23.5 ± 0.8 ^a^
Liver weight (g/100 g body weight)	5.3 ± 0.9 ^a^	5.5 ± 0.5 ^ab^	5.4 ± 0.5 ^ab^	5.6 ± 0.2 ^ab^

Values are expressed as mean ± SD, *n* = 10. a–c Means in a row without a common letter differ, *p* < 0.05.

**Table 3:** Effects of different dose of AEPL on serum biochemical parameters and hepatic TG level in alcohol-induced liver injury in mice.

Groups	Control	EtOH	EtOH + 1% AEPL	EtOH + 2% AEPL
AST (U/L)	169.7 ± 61.2 ^b^	285.8 ± 21.5 ^a^	225.0 ± 53.4 ^ab^	205.0 ± 65.5 ^b^
ALT (U/L)	40.8 ± 6.2 ^b^	65.3 ± 6.1 ^a^	51.8 ± 9.4 ^b^	51.7 ± 8.1 ^b^
TC (mg/dL)	105.7 ± 5.8 ^b^	134.1 ± 17.7 ^a^	121.0 ± 9.5 ^a^	104.6 ± 20.0 ^b^
TG (mg/dL)	67.6 ± 9.4 ^b^	94.1 ± 16.7 ^a^	74.8 ± 13.4 ^b^	73.3 ± 12.1 ^b^
HDL (mg/dL)	49.5 ± 5.6 ^b^	74.5 ± 11.9 ^a^	78.0 ± 12.2 ^a^	69.7 ± 14.5 ^a^
LDL (mg/dL)	40.0 ± 4.6 ^b^	57.0 ± 7.0 ^a^	41.7 ± 5.8 ^b^	45.3 ± 0.6 ^b^
VLDL (mg/dL)	14.8 ± 2.2 ^b^	19.3 ± 3.0 ^a^	15.6 ± 2.5 ^b^	13.3 ± 1.2 ^b^
Hepatic TG (µg/mg protein)	306.3 ± 15.2 ^b^	388.9 ± 33.7 ^a^	360.9 ± 35.4 ^a^	326.4 ± 42.6 ^b^

Values are expressed as mean ± SD, *n* = 10. a,b Means in a row without a common letter differ, *p* < 0.05. AEPL: aqueous extract of pepino leaf; TG: triglyceride; EtOH: alcohol-containing liquid diet group; AST: aspartate aminotransferase; ALT: alanine aminotransferase; TC: total cholesterol; HDL: high-density lipoproteins; LDL: low-density lipoproteins; VLDL: very-low-density lipoproteins.

**Table 4:** Effects of different dose of AEPL on the antioxidant substances and TBARS in alcohol-induced liver injury in mice.

Antioxidant Substances	Control	EtOH	EtOH + 1% AEPL	EtOH + 2% AEPL
SOD (U/mg protein)	1.6 ± 0.3 ^a^	0.8 ± 0.3 ^b^	1.3 ± 0.4 ^a^	1.2 ± 0.4 ^a^
Catalase (U/mg protein)	36.2 ± 7.8 ^ab^	28.5 ± 4.0 ^c^	36.7 ± 5.8 ^b^	45.9 ± 6.2 ^a^
GPx (nmol NADPH/min/mg protein)	32.0 ± 4.4 ^a^	18.2 ± 4.8 ^c^	22.3 ± 6.8 ^bc^	30.1 ± 2.1 ^ab^
GSH (nmol/mg protein)	15.3 ± 2.3 ^b^	17.7 ± 2.6 ^b^	17.1 ± 1.2 ^ab^	20.1 ± 3.1 ^a^
TEAC (mg/dL)	0.18 ± 0.0 ^a^	0.16 ± 0.0 ^b^	0.18 ± 0.0 ^a^	0.19 ± 0.0 ^a^
MDA (nmole/mg protein)	3.2 ± 0.1 ^b^	8.2 ± 1.0 ^a^	2.0 ± 1.0 ^b^	2.0 ± 0.6 ^b^

Values are expressed as mean ± SD, *n* = 10. a–c Means in a row without a common letter differ, *p* < 0.05.

## Supplementary Materials

The following are available online at http://www.mdpi.com/2072-6643/10/7/931/s1. Table S1: Composition of the aqueous extract of pepino leaf (AEPL). Table S2: Composition of the aqueous extract of pepino leaf (AEPL) vs. pepino fruits. Table S3: Comparison of various plant-derived polyphenols.

## References

[1] Gao B., Bataller R. (2011). Alcoholic liver disease: Pathogenesis and new therapeutic targets. Gastroenterology.

[2] Liu J. (2014). Ethanol and liver: Recent insights into the mechanisms of ethanol-induced fatty liver. World J. Gastroenterol..

[3] Li Y., Xu S., Mihaylova M.M., Zheng B., Hou X., Jiang B., Park O., Luo Z., Lefai E., Shyy J.Y. (2011). AMPK phosphorylates and inhibits SREBP activity to attenuate hepatic steatosis and atherosclerosis in diet-induced insulin-resistant mice. Cell Metab..

[4] Rakhshandehroo M., Knoch B., Muller M., Kersten S. (2010). Peroxisome proliferator-activated receptor alpha target genes. PPAR Res..

[5] Birben E., Sahiner U.M., Sackesen C., Erzurum S., Kalayci O. (2012). Oxidative stress and antioxidant defense. World Allergy Organ J..

[6] Jin M., Ande A., Kumar A., Kumar S. (2013). Regulation of cytochrome P450 2e1 expression by ethanol: Role of oxidative stress-mediated pkc/jnk/sp1 pathway. Cell Death Dis..

[7] Lu Y., Cederbaum A.I. (2008). CYP2E1 and oxidative liver injury by alcohol. Free Radic. Biol. Med..

[8] Sid B., Verrax J., Calderon P.B. (2013). Role of oxidative stress in the pathogenesis of alcohol-induced liver disease. Free Radic. Res..

[9] Ayala A., Munoz M.F., Arguelles S. (2014). Lipid peroxidation: Production, metabolism, and signaling mechanisms of malondialdehyde and 4-hydroxy-2-nonenal. Oxid. Med. Cell Longev..

[10] Han K.H., Hashimoto N., Fukushima M. (2016). Relationships among alcoholic liver disease, antioxidants, and antioxidant enzymes. World J. Gastroenterol..

[11] Zhou T., Zhang Y.J., Xu D.P., Wang F., Zhou Y., Zheng J., Li Y., Zhang J.J., Li H.B. (2017). Protective effects of lemon juice on alcohol-induced liver injury in mice. Biomed. Res. Int..

[12] Park H.S., Jung H.Y., Park E.Y., Kim J., Lee W.J., Bae Y.S. (2004). Cutting edge: Direct interaction of TLR4 with NAD(P)H oxidase 4 isozyme is essential for lipopolysaccharide-induced production of reactive oxygen species and activation of NF-kB. J. Immunol..

[13] Chung D.J., Yang M.Y., Li Y.R., Chen W.J., Hung C.Y., Wang C.J. (2017). Ganoderma lucidum repress injury of ethanol-induced steatohepatitis via anti-inflammation, anti-oxidation and reducing hepatic lipid in C57BL/6J mice. J. Funct. Foods.

[14] Tang C.C., Huang H.P., Lee Y.J., Tang Y.H., Wang C.J. (2013). Hepatoprotective effect of mulberry water extracts on ethanol-induced liver injury via anti-inflammation and inhibition of lipogenesis in C57BL/6J mice. Food Chem. Toxicol..

[15] He P., Wu Y., Shun J., Liang Y., Cheng M., Wang Y. (2017). Baicalin ameliorates liver injury induced by chronic plus binge ethanol feeding by modulating oxidative stress and inflammation via CYP2E1 and NRF2 in mice. Oxid. Med. Cell Longev..

[16] Ren W., Tang D.G. (1999). Extract of *solanum muricatum* (Pepino/CSG) inhibits tumor growth by inducing apoptosis. Anticancer Res..

[17] Hsu C.C., Guo Y.R., Wang Z.H., Yin M.C. (2011). Protective effects of an aqueous extract from pepino (*solanum muricatum* Ait.) in diabetic mice. J. Sci. Food Agric..

[18] Ma C.T., Chyau C.C., Hsu C.C., Kuo S.M., Chuang C.W., Lin H.H., Chen J.H. (2016). Pepino polyphenolic extract improved oxidative, inflammatory and glycative stress in the sciatic nerves of diabetic mice. Food Funct..

[19] Yagi K. (1976). A simple fluorometric assay for lipoperoxide in blood plasma. Biochem. Med..

[20] Eyer P., Podhradsky D. (1986). Evaluation of the micromethod for determination of glutathione using enzymatic cycling and ellman’s reagent. Anal. Biochem..

[21] Knight S.A., Sunde R.A. (1987). The effect of progressive selenium deficiency on anti-glutathione peroxidase antibody reactive protein in rat liver. J. Nutr..

[22] Sun Y., Oberley L.W., Li Y. (1988). A simple method for clinical assay of superoxide dismutase. Clin. Chem..

[23] Aebi H., Wyss S.R., Scherz B., Skvaril F. (1974). Heterogeneity of erythrocyte catalase ii. Isolation and characterization of normal and variant erythrocyte catalase and their subunits. Eur. J. Biochem..

[24] Re R., Pellegrini N., Proteggente A., Pannala A., Yang M., Rice-Evans C. (1999). Antioxidant activity applying an improved ABTS radical cation decolorization assay. Free Radic. Biol. Med..

[25] Fowler S.D., Greenspan P. (1985). Application of Nile red, a fluorescent hydrophobic probe, for the detection of neutral lipid deposits in tissue sections: Comparison with oil red O. J. Histochem. Cytochem..

[26] Kim K.H., López-Casillas F., Bai D.H., Luo X., Pape M.E. (1989). Role of reversible phosphorylation of acetyl-COA carboxylase in long-chain fatty acid synthesis. FASEB J..

[27] Brandon-Warner E., Schrum L.W., Schmidt C.M., McKillop I.H. (2012). Rodent models of alcoholic liver disease: Of mice and men. Alcohol.

[28] Pirola R.C., Lieber C.S. (1976). Hypothesis: Energy wastage in alcoholism and drug abuse: Possible role of hepatic microsomal enzymes. AJCN.

[29] Anji A., Kumari M. (2008). Supplementing the liquid alcohol diet with chow enhances alcohol intake in C57 BL/6 mice. Drug Alcohol Depend..

[30] Ji C., Chan C., Kaplowitz N. (2006). Predominant role of sterol response element binding proteins (SREBP) lipogenic pathways in hepatic steatosis in the murine intragastric ethanol feeding model. J. Hepatol..

[31] Horton J.D., Goldstein J.L., Brown M.S. (2002). SREBPs: Activators of the complete program of cholesterol and fatty acid synthesis in the liver. J. Clin. Investig..

[32] Zeng T., Zhang C.L., Song F.Y., Zhao X.L., Xie K.Q. (2014). CMZ Reversed Chronic Ethanol-Induced Disturbance of PPAR-α Possibly by Suppressing Oxidative Stress and PGC-1α Acetylation, and Activating the MAPK and GSK3β Pathway. PLoS ONE.

[33] Crabb D.W., Galli A., Fischer M., You M. (2004). Molecular mechanisms of alcoholic fatty liver: Role of peroxisome proliferator-activated receptor alpha. Alcohol.

[34] Fischer M., You M., Matsumoto M., Crabb D.W. (2003). Peroxisome proliferator-activated receptor α (PPARα) agonist treatment reverses PPARα dysfunction and abnormalities in hepatic lipid metabolism in ethanol-fed mice. J. Biol. Chem..

[35] Klop B., Do Rego A.T., Cabezas M.C. (2013). Alcohol and plasma triglycerides. Curr. Opin. Lipidol..

[36] De Oliveira E.S.E.R., Foster D., McGee Harper M., Seidman C.E., Smith J.D., Breslow J.L., Brinton E.A. (2000). Alcohol consumption raises HDL cholesterol levels by increasing the transport rate of apolipoproteins a-i and a-ii. Circulation.

[37] Fan F., Cao Q., Wang C., Ma X., Shen C., Liu X.W., Bu L.P., Zou Y.Z., Hu K., Sun A.J. (2014). Impact of chronic low to moderate alcohol consumption on blood lipid and heart energy profile in acetaldehyde dehydrogenase 2-deficient mice. Acta. Pharmacol. Sin..

[38] Ambade A., Mandrekar P. (2012). Oxidative stress and inflammation: Essential partners in alcoholic liver disease. Int. J. Hepatol..

[39] Keshavarzian A., Farhadi A., Forsyth C.B., Rangan J., Jakate S., Shaikh M., Banan A., Fields J.Z. (2009). Evidence that chronic alcohol exposure promotes intestinal oxidative stress, intestinal hyperpermeability and endotoxemia prior to development of alcoholic steatohepatitis in rats. J. Hepatol..

[40] Su G.L., Klein R.D., Aminlari A., Zhang H.Y., Steinstraesser L., Alarcon W.H., Remick D.G., Wang S.C. (2000). Kupffer cell activation by lipopolysaccharide in rats: Role for lipopolysaccharide binding protein and toll-like receptor 4. Hepatology.

[41] Kawaratani H., Tsujimoto T., Douhara A., Takaya H., Moriya K., Namisaki T., Noguchi R., Yoshiji H., Fujimoto M., Fukui H. (2013). The effect of inflammatory cytokines in alcoholic liver disease. Mediat. Inflamm..

[42] Da Silva B.S., Rodrigues G.B., Rocha S.W., Ribeiro E.L., Gomes F.O., E Silva A.K., Peixoto C.A. (2014). Inhibition of NF-kB activation by diethylcarbamazine prevents alcohol-induced liver injury in C57BL/6 mice. Tissue Cell.

[43] Bohm F., Kohler U.A., Speicher T., Werner S. (2010). Regulation of liver regeneration by growth factors and cytokines. EMBO Mol. Med..

[44] Isayama F., Froh M., Yin M., Conzelmann L.O., Milton R.J., McKim S.E., Wheeler M.D. (2004). TNF α-induced ras activation due to ethanol promotes hepatocyte proliferation independently of liver injury in the mouse. Hepatology.

[45] Zhang X., Tachibana S., Wang H., Hisada M., Williams G.M., Gao B., Sun Z. (2010). Interleukin-6 is an important mediator for mitochondrial DNA repair after alcoholic liver injury in mice. Hepatology.

[46] Kumar S., Pandey A.K. (2013). Chemistry and biological activities of flavonoids: An overview. Sci. World J..

[47] Zhu W., Jia Q., Wang Y., Zhang Y., Xia M. (2012). The anthocyanin cyanidin-3-O-β-glucoside, a flavonoid, increases hepatic glutathione synthesis and protects hepatocytes against reactive oxygen species during hyperglycemia: Involvement of a cAMP-PKA-dependent signaling pathway. Free Radic. Biol. Med..

[48] Ludwig A., Lorenz M., Grimbo N., Steinle F., Meiners S., Bartsch C., Stangl K., Baumann G., Stangl V. (2004). The tea flavonoid epigallocatechin-3-gallate reduces cytokine-induced VCAM-1 expression and monocyte adhesion to endothelial cells. Biochem. Biophys. Res. Commun..

[49] Pezeshki A., Safi S., Feizi A., Askari G., Karami F. (2016). The effect of green tea extract supplementation on liver enzymes in patients with nonalcoholic fatty liver disease. Int. J. Prev. Med..

