# Supplementary material for: Aqueous Extract of Pepino (Solanum muriactum Ait) Leaves Ameliorate Lipid Accumulation and Oxidative Stress in Alcoholic Fatty Liver Disease
Source: Nutrients. 2018 Jul 20;10(7):931. doi: 10.3390/nu10070931 (PMC6073701; doi:10.3390/nu10070931)
Supplement: Supplementary file 1 [file nutrients-10-00931-s001.pdf]

nutrients

Table S1. Composition of the aqueous extract of pepino leaf (AEPL).

Polyphenolic compound	AEPL[a] (%)
Total phenolic acid (Folin-Ciocalteu method [b])	5.52 ± 1.27
Total flavonoids (Jia method[c])	25.32 ± 0.48
Total anthocyanins (Fuleki and Francis method[d])	2.02 ± 1.13

[a] The AEPL was prepared as described in Materials and Methods. [b-d] The concentrations of total phenolic accid, flavonoid, and anthocyanin were analyzed according to the Folin-Ciocalteau method [Lakenbrink et al., 2000], Jia method [Jia et al., 1998], Fuleki and Francis method [Fuleki and Francis, 1968], receptively.

Table S2. Composition of the aqueous extract of pepino leaf (AEPL) *vs.* pepino fruits.

**Polyphenolic compounds (mg/100g dry weight)**	**AEPL[a]**	**PAE [b]**
Total phenolic acid (Folin-Ciocalteu method [c])	933 ± 228	1217 ± 188
Total flavonoids (Jia method [d])	4550 ± 86	875 ± 62
Total anthocyanins (Fuleki and Francis method [e])	360 ± 203	177 ± 51

a The AEPL was prepared as described in Materials and Methods.b The composition of aqueous extract of pepino fruit (PAE) was cited by the study of Hsu, Guo et al.**c**-e The concentrations of total phenolic acid, flavonoid, and anthocyanin were analyzed according to the Folin-Ciocalteau method (Lakenbrink et al., 2000), Jia method (Jia et al., 1998), Fuleki and Francis method (Fuleki and Francis, 1968), receptively.

Table S3. Comparison of various plant-derived polyphenols.

		AEPL	HSE	GTE
Full name		aqueous extract of pepino leaf	aqueous extracts from *Hibiscus sabdariffa*	Green tea extract
Component		phenolic acids, flavonoids, anthocyanins,	phenolic acids, flavonoids, anthocyanins	EGCG, EGC, ECG, EC, caffeine
Major compound (%)		N/A [a]	N/A	EGCG (30–42%)
Anti-cancer and oxidative DNA damage	Animal models	N/A	1–2% (ICR mice)	2% (A/J mice)
	Human studies	N/A	N/A	3 g/day; 10%
Anti-lipid peroxidation and atherosclerosis	Animal models	N/A	0.5% –1% (New Zealand White rabbits)	50 mg/kg; 3% (Wistar rats)
	Human studies	N/A	1 g/day	600 mg/day
Hypoglycemic	Animal models	N/A	200 mg/kg (SD rats)	500 mg/kg (SD rats)
	Human studies	N/A	2 g/day	857 mg/day
Hepatoprotection	Animal models	1–2% (C57BL/6J mice)	1–5% (Wistar rats); 200–600 mg/kg (BALB/c mice)	2.5% (Wistar rats)
	Human studies	N/A	N/A	N/A
Reference		In our manuscript, 2017	Chen et al., 2005; Lin et al., 2007; Liu et al., 2006; Liu et al., 2010; Mozaffari-Khosravi et al., 2008; Pheng et al., 2011	Higdon & Frei, 2003; Hsu et al., 2011; Kuzu et al., 2008; Lin et al., 1998; Li, Sun, Han, & Chen, 1999; Miura et al., 2000; Sano etal., 1995; Xu et al., 1992

N/A, Not available.
